# Hypercoagulability in Light Chain Amyloidosis and the Importance of Predictive Value of TEG and TGT for Thrombosis Recurrence in Inflammatory States

**DOI:** 10.3390/diagnostics16070987

**Published:** 2026-03-25

**Authors:** Mihai Emanuel Himcinschi, Mihaela Uta, Andreea Jercan, Daniel Murariu, Delia Codruta Popa, Valentina Uscatescu, Andrei Anghel, Daniel Coriu, Sorina Nicoleta Badelita

**Affiliations:** 1 Haematology and Bone Marrow Transplant Centre, Clinical Institute Fundeni, 022328 Bucharest, Romania; mhimcinschi@gmail.com (M.E.H.); je.andreea@gmail.com (A.J.); popadeliacodruta@gmail.com (D.C.P.); daniel_coriu@yahoo.com (D.C.); sorinabadelita@gmail.com (S.N.B.); 2 Haematology Department, University of Medicine and Pharmacy Carol Davila, 020021 Bucharest, Romania; 3 Department of Biochemistry, Victor Babes University of Medicine and Pharmacy, 300041 Timişoara, Romania

**Keywords:** light chain amyloidosis, hypercoagulability, thrombosis, inflammation, specialized coagulation assays, thromboelastography (TEG), thrombin generation time (TGT)

## Abstract

**Background**: Thrombosis in light chain amyloidosis (LCA) occurs in the context of multiple organ dysfunction and inflammation. Conventional coagulation tests (screening) may not sufficiently capture the procoagulant substrate in the inflammatory/therapeutic dynamics. **Methods**: A total of 61 consecutive patients with LCA were prospectively included in the study. Clinical data, including organ involvement, time of diagnosis, treatment phase, DOAC exposure and thrombosis history were systematically recorded and subjected to screening. Specialized hemostasis tests such as APTT/PT, fibrinogen, D-dimer, TEG and TGT were performed and conventional times were analyzed in the subgroup without DOAC. **Results**: The prevalence of documented thrombosis was 32.8%, and thrombosis status was associated with TEG positivity and more strongly with TGT positivity. Hypercoagulability was identified in 50.8% by TEG and 41.0% by TGT, regardless of whether APTT/PT were within the reference values. APTT/PT did not predict thrombosis recurrence (*p* > 0.05), which was predicted by TEG (*p* = 0.0027) and TGT (*p* = 0.0006). An inflammation/fibrin turnover panel (CRP, fibrinogen, D-dimer) predicted TEG positivity (*p* < 0.0001), but not TGT, and was correlated with assessment at diagnosis, daratumumab-based therapy, and cardiac involvement. **Conclusions**: Global tests (TEG/TGT) promptly correlate with thrombosis recurrence in our cohort and provide crucial information in addition to clotting times for thrombotic phenotyping. Inflammation can influence TEG, so the decision to recommend the tests and the timing of their performance should be adapted to the clinical, biological, and therapeutic context.

## 1. Introduction

Light chain amyloidosis (LCA) is a systemic protein misfolding disorder in which immunoglobulin light chains formed by clonal plasma cells are deposited as insoluble amyloid fibrils, generating progressive malfunction of various organs and tissues [1,2,3]. Even though all organs may be involved, the kidneys and heart are the most common sites involved in LCA [4]. Renal involvement is the most frequent manifestation, while cardiac involvement remains the main determinant of patient survival and prognosis [2]. Other organs, like the liver and gastrointestinal tract, may also be involved, giving LCA an intricate and highly divergent clinical profile [5]. Some of the less reported complications of LCA are the association of hemostatic abnormalities that could impact the disease progression [1]. Even though hemorrhagic implications are generally better characterized than thrombotic complications [6], the literature suggests that coagulation test abnormalities are relatively common in clinical practice [7].

In addition to bleeding complications, LCA is increasingly recognized as a clinical framework in which thrombotic manifestations take place with a clinically consistent frequency; however, the thrombotic risk remains incompletely defined [7,8,9]. The prothrombotic status in LCA is likely multifactorial and includes acute phase transitions mediated by endothelial dysfunction, immobilization, heart failure, inflammation in advanced forms of heart disease, urinary loss of proteins with depletion of natural anticoagulants, as well as factors connected to treatment or changes in vascular architecture given by amyloid deposition [7]. The production of neutrophil extracellular traps (NETs) should also be considered in the case of prothrombotic events in highly inflammatory contexts [10]. NETs, resulting from the proinflammatory and endothelial-dysfunctional environment in LCA, may contribute to thrombosis and organ damage [11]. Simultaneously, LCA could also predispose to bleeding complications, which could be explained by underlying mechanisms such as vascular fragility, acquired coagulation factor deficiencies, and thrombopathies, resulting in an intricate and often paradoxical hemostatic phenotype [12].

A major challenge in clinical practice is that conventional coagulation tests provide only a partial and static picture of coagulation and may not adequately reflect the dynamics of the whole blood clot or the overall thrombin-forming capacity [13]. Consequently, we are starting to see growing endorsement for global hemostasis lab work, which includes viscoelastic explorations and thrombin generation testing, which contribute complementary as well as functional information [14,15,16]. Thromboelastography (TEG) integrates cellular as well as plasmatic coagulation attributes and provides a systematic characterization of clot initial formation, strength, and propagation [17], while thrombin generation time (TGT) quantifies the capacity of plasma to form thrombin over time [18], the central element in fibrin formation and, therefore, thrombosis. However, prospective articles evaluating how these explorations contribute to the prothrombotic status in LCA remain limited, particularly in more recent cohorts with exposure to heterogeneous therapeutic approaches and frequent use of DOACs (direct oral anticoagulants), which may influence, in some circumstances, test interpretation.

In this context, we performed a prospective, single-centre study to characterize hemostatic and inflammatory profiles in LCA using a structured panel of biochemical markers, conventional hemostasis parameters (including indices of clotting time and factor X activity (FX), carefully interpreted in the presence of anticoagulant exposure), and global functional testing by TEG and TGT. The primary objective was to assess whether global hemostatic phenotypes are associated with a thrombotic clinical profile in LCA and to explore the relationship between hypercoagulability signatures and markers related to inflammation, organ involvement, and treatment context.

## 2. Materials and Methods

### 2.1. Ethics

This study was conducted within the Department of Hematology of the Fundeni Clinical Institute, Bucharest, in accordance with the Declaration of Helsinki and its amendments. All patient data were anonymized before statistical analysis and interpretation of the results. The protocol of this study was approved by the hospital ethics committee. Informed consent was obtained for participation in the study at the time of diagnosis or at the patient follow-up visit, in accordance with the procedural standards of the Institute.

### 2.2. Patients and Study Design

This prospective, single-centre study investigated hemostasis and coagulation profiles in a cohort of 61 consecutive patients with confirmed light chain amyloidosis, who were enrolled and evaluated at our institute between January 2024 and June 2025.

Clinical and laboratory data relevant to thrombotic risk and hemostatic status were collected in the study, including previous thrombotic history, major organ involvement, current disease status, antithrombotic therapies, background therapy, ongoing therapy, albumin levels, kappa/lambda (*κ*/*λ*) light chains, C-reactive protein (CRP), global hemostasis tests such as prothrombin time (PT), activated partial thromboplastin time ratio (APTT-R), fibrinogen (FG), D-dimers specialized hemostasis tests (TEG, TGT, FX).

Major organ involvement was systematically recorded (dichotomously), thus documenting patients who had renal, cardiac, or hepatic involvement. These organ involvements were selected because organ dysfunction related to LCA may influence hemostasis through their distinct mechanisms.

Global coagulation status was documented using viscoelastic functional tests, as well as plasma-based tests (APTT, PT, fibrinogen, factor X). The TEG-derived hypercoagulability classification was documented to provide an integrative, whole blood-based assessment of clot initiation, strength, stability, and kinetics. In parallel, we documented the thrombin generation (TGT)-derived hypercoagulability classification, as thrombin generation represents a central mechanistic readout of coagulation potential and can sensitively detect thrombosis-relevant procoagulant changes in plasma systems [19,20].

Finally, treatment-related parameters at the time of patient inclusion were recorded to account for how they may influence laboratory results. This included mention of background therapy, with particular attention to daratumumab-based therapeutic protocols, thus taking into account the potential of therapy to modify disease activity, systemic inflammation, endothelial activation, and ultimately the hypercoagulability profiles given by laboratory results [21,22,23]. It was also documented whether data recording occurred at initial diagnosis versus during treatment. In addition, the use of direct oral anticoagulants (DOACs) at the time of sample collection was documented to allow for accurate interpretation of test results.

### 2.3. Sample Processing

Biochemistry assays (albumin, free light chains κ and λ, CRP) were accomplished using the patient’s serum. Venous blood was gathered in serum separator tubes using the accepted aseptic techniques, with reduced application of the tourniquet. Samples were deposited to clot for a minimum of half an hour, following centrifugation and processing within 2 h of collection (2500× *g*, 10 min). Serum was separated immediately after centrifugation and processed or stored (at 2–8 °C for up to 72 h or at −20 °C for long-term storage). Samples that showed macroscopic hemolysis, icterus or lipemia were excluded from the analysis, regardless of the sample type. Serum albumin was determined by a colorimetric method using bromocresol green and bromocresol purple on an open automated biochemistry platform, and C-reactive protein (CRP) was measured on the same platform by immunoturbidimetric methods. Serum free κ and λ light chains were measured using automated immunonephelometry or immunoturbidimetry. Calibration and internal quality controls were performed according to the manufacturer’s recommendations for each assay.

Venous blood for coagulation tests was collected in 3.2% sodium citrate tubes (anticoagulant-blood ratio 1:9). The tubes were gently shaken to mix with the anticoagulant and analyzed for micro-clots. Platelet-poor plasma (PPP) was collected by centrifugation at 2500× *g* for 15 min then carefully separated without disturbing the top cell layer. Plasma was analyzed promptly, when possible, otherwise, 1–2 mL aliquot tubes were stored for short periods of time at −20 °C or long term at −80 °C, then defrosted at 37 °C before testing. Coagulated, underfilled, or hemolyzed samples were excluded from the workflow.

Activated partial thromboplastin time, as well as prothrombin time (APTT and PT) were made using an automated open platform analyzer (Stago R-MAX III) using reagents regulated by the manufacturer [24]. Results were expressed as ratio for APTT and percentage for PT. Fibrinogen was determined using the Clauss method, and plasmatic D-dimers levels were assessed by immunological methods [24,25]. Factor X activity was performed using a one-stage coagulation test, and the activity was reported as a percentage out of normal.

Viscoelastic testing was performed by TEG on fresh whole blood (standard collection on sodium citrate), according to the manufacturer’s protocol (additional activation with kaolin), reporting R, K time, ANGLE, maximum amplitude (MA) and lysis indices [26]. This method was used as a screening test for hypercoagulability as TEG parameters are more sensitive to FG levels (especially the MA).

Thrombin generation was assessed in platelet-poor plasma using the automated Stago-Genesia analyzer. Lag, time to peak, peak and total endogenous thrombin potential were derived from the thrombin generation curve [27]. Calibration, internal quality controls and participation in external quality assessment were applied throughout the testing, and all analyses were performed in accordance with the standard operating procedures of the internal laboratory and the manufacturer’s instructions and legal regulations related to diagnostic propaedeutics.

### 2.4. Statistical Analysis

Statistical workflow was performed with GraphPad Prism for Windows (v.10) software (GraphPad Software, San Diego, CA, USA).

Given the possibility of a non-Gaussian distribution of coagulation results in clinical cohorts and the rather small to medium sample size, continuous variables were briefed using the interquartile range (IQR/25th–75th percentiles) and median. Where relevant to characterize extreme values, the 95th percentile (P95) was additionally reported.

Correlations between dichotomous variables were evaluated using Fisher’s exact test (with Monte Carlo approximation).

To investigate independent relationships between laboratory predictors and binary outcomes (e.g., presence or absence of a particular clinical feature), multiple logistic regression was applied. The multiple logistic regression analysis test is used when the outcome is documented as binary (examples: yes/no, present/absent). In order to explain/predict this outcome at least two predictors (continuous independent variables in our case) are used. The core idea of the test is that instead of modelling the outcome directly, it models the log-odds of the outcome being 1 then converts the linear score into a probability using a logistic S-shaped mathematical function. Throughout the article the concept of “model”, “multivariable analysis” or “logistic model” will be used as a reference to the functionality of the statistical test (the different outcomes and predictors used each time the test was used will also be specified). The outcomes of the multiple logistic regressions were quantified as *p*-values, along with the area under the curve (AUC). A *p* value < 0.05 was considered statistically significant.

## 3. Results

### 3.1. Patient Characteristics

Throughout the cohort, 20 participants (32.8%) had thrombosis in their history, of which six participants (9.8%) presented thrombosis before the diagnosis was confirmed. Organ involvement was confirmed as follows: hepatic involvement in 21 patients (34.4%), renal involvement in 34 patients (55.7%), and cardiac involvement in 37 participants (60.7%), of which 20 patients (32.7% out of total participants) were documented with atrial fibrillation and 17 patients (27.8% out of total participants) were documented with heart failure (categories are not mutually exclusive). Hemostasis and coagulation assays identified a hypercoagulable phenotype for 31 participants (50.8%), using TEG and for 25 participants (41.0%) using TGT. Regarding therapeutic status at the time of patients inclusion in the study, 43 participants (70.5%) were receiving daratumumab-based therapy, 18 participants (29.5%) were included at the initial diagnosis of the disease, and 26 participants (42.6%) were under anticoagulation therapy using DOACs, most of them for prophylactic purposes for atrial fibrillation (20 patients—32.7%). In order to evaluate disease prognosis/severity, the Palladini and Mayo staging are presented alongside patient characteristics in Table 1.

Within the cohort, the biochemical profile is characterized by a median serum albumin level of 3.6 g/dL (IQR 2.8–9.8), along with a right-skewed distribution of free light chains k, 16 mg/L (IQR 8.2–59) and l, 23 mg/L (IQR 12–48) and an increased inflammatory status, with CRP 8 mg/L (IQR 2.7–24). In the subgroup that did not receive DOACs, coagulation parameters were generally within reference ranges, with APTT ratio 0.97 (IQR 0.92–1.12), PT 96% (IQR 84–109), and FX 98% (IQR 78–156), while the broader haemostasis panel indicated an inflammatory increase in fibrinogen (490 mg/dL, IQR 396–570) and a D-dimer distribution with a median below the cut-off threshold (224 ng/mL, IQR 132–457) but a significantly increased upper end, 95th percentile (P95, 1118 ng/mL). Whole blood TEG testing demonstrated values consistent with preserved clot initiation kinetics (R 5.3 min, K 1.2 min) and a trend toward stronger and faster clot formation in the upper part of the graph, with an ANGLE of 73°, MA 68 mm, and G 10. Thrombin generation (TGT) additionally showed a wide dispersion, with median LAG of 3.7 min, peak of 175 nM, time to peak of 6.7 min, and ETP of 1127 nM/min, and maximum 95th percentiles (11 min, 470 nM, 12 min, and 2169 nM/min, respectively), supporting substantial interindividual variability in thrombin generation capacity within the cohort. All of the results above (medians, IQR, as well as the normal interval ranges, in addition to the 95% percentile) are displayed in Table 2.

Table 2: κ and λ—light chains kappa and lambda; TEG-derived parameters are composed by: R (reaction time—the distance from the start of the sample run to the point of the first clot formation measured in minutes), K (distance required for the clot strength to reach an amplitude of 20 mm, measured in minutes), ANGLE (the measurement in degrees of arc from the point of divergence to the maximum tangent drawn to the curve of the clot formation), MA (the maximum amplitude of the clot strength prior to clot lysis, influenced by fibrinogen and platelet activity, measured in mm), G (the shear elastic modulus strength of the clot measured in d/sec); TGT-derived parameters: LAG (the time necessary for the reaction to start—the latency period before the thrombin generation begins measured in minutes), PEAK (the maximum thrombin level that has been reached measured in nM), TIME TO PEAK (the time between the debut of thrombin generation and the PEAK measured in minutes), ETP (the area under the thrombin generation curve that represents the total endogenous thrombin generation potential measured in nM/min).

### 3.2. Correlation Between Thrombosis Recurrence and Organ Involvement, Moment of Diagnosis and Therapeutic Status

In order to document whether thrombosis recurrence could be associated with determinant clinical variables, a series of Fisher’s exact tests were performed, that compare thrombosis history with major organ involvement, presence of treatment, and diagnostic moment. No significant statistical correlations were identified between thrombosis and the moment of patient presentation at initial diagnosis (*p* > 0.05) or being under daratumumab-based therapy at the time of the inclusion in the study (*p* > 0.05). Similarly, thrombotic recurrence was not statistically associated with renal involvement (*p* > 0.05), hepatic involvement (*p* > 0.05), or cardiac involvement (*p* > 0.05). Collectively, these analyses did not show a notable relationship between thrombotic recurrence and the documented patterns of treatment status, organ involvement, or timing of diagnosis in our cohort.

### 3.3. Correlation Between Thrombosis Recurrence and Test-Dependent Hypercoagulability Status, Their Utility as Prediction Factors and How Inflammation Impacts Thromboelastography and Thrombin Generation Time Results and Interpretation

**Figure 1:**
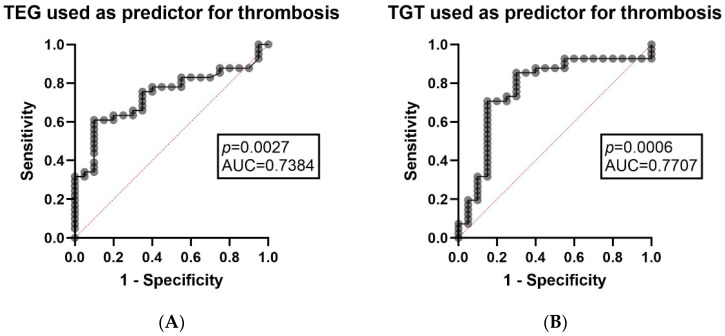
TEG and TGT parameters predicting thrombosis recurrence. (**A**)—K, ANGLE, and MA (TEG-derived parameters) used as predictors in order to determine the thrombosis recurrence (outcome) using the multiple logistic regression analysis (left); (**B**)—Peak and ETP (TGT-derived parameters) used as predictors in order to determine the thrombosis recurrence (outcome) using the multiple logistic regression analysis (right).

Subsequently, multivariable analyses were performed to assess predictors for thrombosis recurrence and to explore determinants of hypercoagulability, as defined by global tests. Using the multiple logistic regression analysis in order to predict the thrombosis recurrence (outcome) using variables (predictors) that include APTT ratio and prothrombin time (PT), we conclude that conventional clotting times did not significantly predict thrombosis recurrence (*p* > 0.05). In contrast, the same statistical analysis that uses TEG parameters (K, ANGLE, and MA) as predictors was significantly associated with thrombosis recurrence (*p* = 0.0027, Figure 1A), indicating that whole blood viscoelastic markers of clot kinetics and clot resistance were predictive of thrombotic status in this cohort. Similarly, using a multiple logistic regression model that uses TGT parameters (Peak and ETP) as predictors, we conclude that thrombin generation capacity was much more strongly related to thrombosis recurrence (*p* = 0.0006 Figure 1B).

Consistent with these results, contingency table analyses proved significant correlations between thrombosis history and hypercoagulability status put into perspective by our viscoelastic and thrombin generation assays. Fisher’s exact test showed that thrombosis status was associated with TEG-defined hypercoagulability positivity (*p* = 0.0066) but even more strongly with TGT-defined hypercoagulability positivity (*p* < 0.0001).

**Figure 2:**
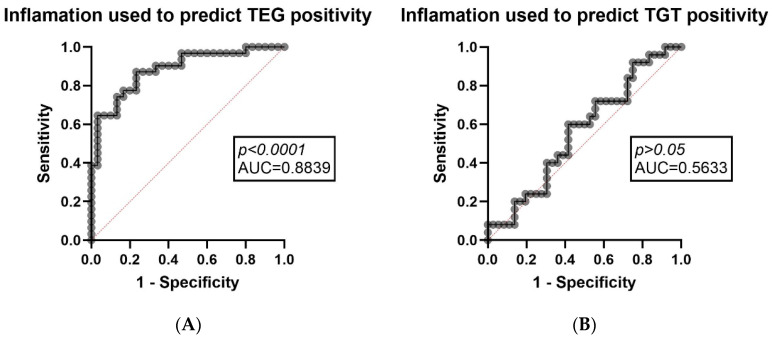
Inflammation markers predicting hypercoagulation in TEG and TGT. (**A**)—CRP, FG and D-dimer (inflammation derived parameters) used as predictors in order to determine TEG positivity for hypercoagulation (outcome) using the multiple logistic regression analysis (left); (**B**)—CRP, FG and D-dimer (inflammation derived parameters) used as predictors in order to determine TGT positivity for hypercoagulation (outcome) using the multiple logistic regression analysis (right).

Finally, we determined whether systemic inflammation and fibrin turnover level markers were related to the hypercoagulability status shown by our global testing. Using multiple logistic regression, a multivariable model that uses CRP, FG, and D-dimer as predictors, positively predicted the outcome defined as TEG-positive status for hypercoagulability (*p* < 0.0001). In contrast, the analogue model that uses the same multiple logistic regression analysis principle did not significantly predict the outcome of TGT-positive status for hypercoagulability, using Peak and ETP variables as predictors (*p* > 0.05), suggesting that, within this data set, parameters related to inflammation were more closely aligned with viscoelastic hypercoagulability investigations rather than with positivity defined by plasma thrombin generation testing. These findings are consistent with the platform-based differences between TEG and TGT, as TEG results are known to be highly dependent on fibrinogen levels (especially the MA of the clot strength prior to clot lysis influenced by fibrinogen and platelet activity). The results are shown in Figure 2A,B.

### 3.4. Inflammatory Stratification in Light Chain Amyloidosis

**Figure 3:**
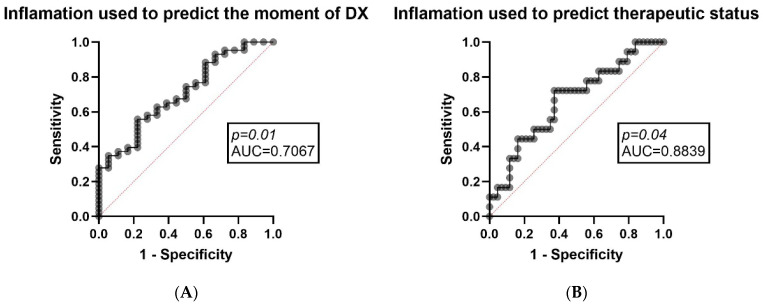
Inflammation markers predicting the moment of study inclusion and therapy status. (**A**) CRP, FG and D-dimer (inflammation derived parameters) used as predictors in order to determine if the patient was included in the study at the moment of diagnosis (outcome) using the multiple logistic regression analysis (left); (**B**) CRP, FG and D-dimer (inflammation derived parameters) used as predictors in order to determine if the patient was under daratumumab-based therapy at the moment of inclusion in the study (outcome) using the multiple logistic regression analysis (right).

Multiple logistic regression was used to assess if inflammation markers and plasmatic levels of fibrin (CRP, D-dimer, and fibrinogen) can be used as predictors of specific clinical framework outcomes. The results accurately predicted the outcome if the patients were investigated at initial diagnosis (*p* = 0.0114) and also promptly predicted the outcome if they were under any active daratumumab-based therapy at the time of their inclusion in the study (*p* = 0.0414). When organ involvement was analyzed, using the same set of lab work, a borderline association with liver involvement (*p* = 0.0539) was established, while there was a significant association with cardiac involvement (*p* = 0.0131). These results indicate that the global inflammatory profile captured by CRP, D-dimer, and fibrinogen varies with disease progression and is more promptly related to cardiac involvement rather than to hepatic involvement in our cohort. The results are shown in Figure 3A,B.

## 4. Discussion

The presented cohort reflects a defining population for how laboratory explorations of blood coagulation overlap with a group of patients with multiple organ involvement, anticoagulant medication, and complex inflammatory processes. A defining feature is the substantial impact of major organ involvement, especially cardiac (60.7%) and renal (55.7%), with hepatic involvement present in 34.4%, which confers a real clinical impact because organ dysfunction is closely linked to hemostatic balance through multiple pathways [28,29,30]. Hepatic function of coagulation factor synthesis [31], renal protein loss [32], and the severity of systemic disease that is captured in cardiac involvement [33] are just a few examples of the mechanisms by which exploratory propaedeutics assess the risk of bleeding or thrombosis, and they must be constantly adapted to ensure consistent accuracy. Consequently, the impact of the study is relevant to similar LCA populations, given the prevalence and low incidence of the disease type.

Another defining feature of the population is the high prevalence of documented thrombosis (32.8%), underlining that thrombotic complications are not rare events in the clinical trajectory of the patient with LCA and supporting the rationale for intensive haemostatic phenotyping. This prevalence is higher than previously reported results in other series and retrospective analyses on thrombotic risk, where thrombosis rates in LCA are often described as single-digit percentages [34]. These differences may be due to variability in study designs and outcome measures, or even due to the size of the cohort to be analyzed. In this context, the prevalence of thrombosis observed in our cohort most likely reflects an interaction of lifestyle-related pathologies (patients with multiple organ involvement superimposed on a logistic space known for a high prevalence of diseases in the cardiovascular domain), together with a more extensive inclusion of thrombotic history documentation.

Finally, the therapeutic landscape of the cohort is contemporary and useful to explicitly incorporate into the interpretation of both clinical associations and the way in which diagnostic reasoning of hemostasis and coagulation studies occurs. The majority of patients were receiving daratumumab-based therapy (70.5%), almost a third were assessed at initial diagnosis (29.5%), and a large proportion were on DOAC anticoagulant therapy (42.6%), mostly preventive (atrial fibrillation), but also in curative doses after a thrombotic event. These characteristics are not merely descriptive; plasma cell-targeted therapy may influence systemic inflammation [35] and endothelial activation over time, while DOAC exposure introduces a major analytical and biological confounder for routine testing in the context of thrombotic risk assessment [36]. Therefore, our analytical decision to interpret selected conventional coagulation parameters in the DOAC-free subgroup is methodologically appropriate, but residual confounding factor remains a key consideration for interpreting screening tests in everyday practice for patients under DOAC therapy, in centres with no access to specialized investigations (TGT).

In the study population, a central finding is the high prevalence of global hypercoagulability, documented in 50.8% of patients by TEG and 41.0% by TGT, indicating that a functional ex vivo procoagulant phenotype is frequently encountered in our cohort compared with screening tests. A second major issue of discussion is the limited predictive value of conventional clotting time tests for thrombotic phenotypes, in contrast to the stronger feedback provided by specialized tests. In our multivariable model, the APTT and PT ratios did not predict recurrent thrombosis (*p* > 0.05), suggesting that clotting-time-based tests, which mainly reflect early plasma clot initiation, may be insufficient to capture the prothrombotic biology found in our pathophysiological context. This supports the premise that conventional hemostasis testing is not sufficient to capture coagulation-relevant changes [37] and also raises the methodological point that TEG and TGT are not interchangeable [38]. They interrogate different biological compartments (whole blood vs. platelet-poor plasma) and different aspects of coagulation dynamics. The wide dispersion observed between TEG versus TGT parameters in Table 2 is consistent with substantial interindividual heterogeneity, which may translate into partial discordance in the classification of “positivity” between platforms, even when both signal hypercoagulability at the study participant level.

This interpretation is also supported by the fact that, even when conventional parameters are largely within reference ranges in the non-DOAC subgroup, the studied cohort still demonstrates frequent hypercoagulability by global functional methods, suggesting that the predisposition to thrombosis may be more closely explained by clot propagation and the ability of plasma to generate thrombin than by initiation times alone. Finally, the clinical relevance of global testing is strengthened by the correlation between hypercoagulability and thrombosis and by the superior discriminative relevance of plasma thrombin generation. Specifically, recurrent thrombosis in the analyzed population was significantly associated with TEG positivity (*p* = 0.0066) and even more strongly with TGT positivity (*p* < 0.0001).

In parallel, prediction models using TEG parameters (MA, ANGLE, K) (*p* = 0.0027) and TGT parameters (ETP, Peak) (*p* = 0.0006) were able to predict thrombosis recurrence, suggesting a pathophysiological interpretation in which both kinetics, the proper structure of the whole blood clot, including the potential of plasma-generated thrombin contribute to the described thrombotic phenotype. The type of organ involvement documented or the patient’s medical evolution might be considered plausible factors that could facilitate thrombosis [2,33]. However, in this cohort, our statistical analysis did not detect a significant association between the recurrence of thrombosis and a specific type of organ involvement, whether the patient was receiving daratumumab-based therapy or if the patient was included in the study at the time of initial diagnosis.

These results can be interpreted in accordance with the documented organ involvement as a binary variable, but may not capture sufficient severity stratifications that are more directly related to thrombotic biology (protein loss in the nephrotic interval with concomitant increase in lipoprotein production, advanced cardiac dysfunction with recurrent blood flow disturbance and endothelial-haemostatic balance disturbances), while therapeutic variability and direct oral anticoagulant therapy may influence the differences observed between groups. In addition, the cohort size, considered to be small to medium for the type of pathology chosen to be studied, and the distribution of events may limit the relevance of the statistical results for detecting associations and how statistical correlations occur in stratified contingency tables.

A significant finding is the platform-specific association (used for biological product analysis—whole blood vs. plasma—TEG vs. TGT) between inflammation/fibrin production and hypercoagulability status. In our multivariable model, the combined set of selected inflammatory markers (D-dimers, FG and CRP) strongly predicted how they influence a positive result on the TEG platform (*p* < 0.0001), while the same model did not influence the interpretability of the result on the TGT platform (*p* > 0.05). This propaedeutic divergence supports a discussion related to the mechanism of the platform used to stratify the result [31], according to which the positivity of viscoelastic hypercoagulability of whole blood is more strongly correlated with acute phase biology (namely, the fact that inflammation plays a much more responsive/determining role for fibrinogen-mediated clot firmness and cellular contributions of platelets, red blood cells or even neutrophils) [39] than the positivity stratified by the generation (or generation potential) of plasma thrombin [40], which may be influenced by a more sensitive balance of anticoagulant and procoagulant forces and may be more susceptible to preanalytical or pharmacological factors. The explanation resides in the way fibrinogen defines the maximum amplitude parameter on TEG as MA is known to be influenced by FG and platelet activity and is the main parameter used for hypercoagulability appreciation. In practical terms, these results substantiate the utility of TEG testing and complementary TGT (in case TEG is positive for a hypercoagulability status), with exclusivity interpreted in the clinical and biological context, in order to exclude the bias factor induced by inflammation on TEG, the positivity of hypercoagulability on TEG being more easily aligned with inflammation.

Finally, the results of inflammation stratification, based on the multivariable model used, provide a coherent anchor for interpreting the impact of inflammation in our cohort. The same panel of markers (CRP, FG, D-dimers) was able to significantly predict whether patients were assessed at diagnosis (*p* = 0.0114) or whether they were on treatment (*p* = 0.0414), and this correlation was significantly associated with cardiac involvement (*p* = 0.0131), while showing only a borderline association with hepatic involvement (*p* = 0.0539). These findings can be assimilated into clinical practice in that the inflammatory/fibrin turnover environment in LCA is not static, but varies according to the phase of the disease (often patients present at diagnosis with an acute event) or the existence of treatment (known to support the inflammatory status) and seems more closely related to cardiac involvement [7,34]. Conceptually, this supports the consideration that parameters used to explore inflammation and fibrin turnover may have a potential use as markers of systemic burden. This information could be integrated as part of daily medical practice and may modulate the dynamics of whole blood laboratory tests, even when they do not translate into a uniform impact on plasma thrombin generation.

A feasible workflow in daily clinical practice can be represented by the recommendation of global testing (TEG/TGT) at key decision points, especially at diagnosis and during monitoring during treatment, given that markers of inflammation/fibrin turnover vary according to the phase of the disease or treatment and predict TEG positivity in our data set. TEG may provide earlier prospective insight and shorter follow-up times, depending on a less laborious technique, but it is imperative to assess the inflammatory status before referral for testing. If the inflammatory status denotes an acute process, the propaedeutics can be directed towards testing for thrombin generation. Implementation should also explicitly take into account DOAC exposure and the time of sampling, consistent with the methodological separation of the selected conventional tests in the non-DOAC subgroup.

Strengths of this article include the prospective design, systematic capture of key clinical variables, and global dual-platform testing (TEG and TGT) with defined preanalytical steps and quality procedures. The analytical approach appropriately combined Fisher’s exact test with multivariable predictive models for dichotomous endpoints.

Limitations are mainly driven by the size of the cohort to be analyzed and endpoints, namely the single-centre setting with a moderate sample size (*n* = 61), heterogeneity of disease stage and therapy, substantial exposure to alternative oral anticoagulants (DOACs), and the use of history of thrombosis rather than incident events that may be inducing or facilitating thrombotic events. Next steps should prioritize prospective validation of incident events, longitudinal sampling for each study participant (from diagnosis to response to treatment), standardized timing with respect to anticoagulants, and use of the proposed workflow for its validation. Another important step should also include the expansion of the cohort to a more statistically significant number, focusing on the uniformization of the subgroups so that the statistical analysis provides results that could be presented as conclusions, and not as suggestions, regarding the predictive value of our variables.

## 5. Conclusions

This prospective cohort addressing LCA captures a complex population in which multiorgan dysfunction (cardiac, renal, and hepatic) and plasma cell-directed therapy influence the interpretation and exploratory recommendations of hemostasis and coagulation. Because the aforementioned factors modify the way we classically interpret laboratory test results, the assessment of thrombosis risk must be individualized.

Thrombosis was frequently documented, supporting the hypothesis of extensive phenotyping. Global functional tests showed frequent hypercoagulability (TEG and TGT) even when clotting times were recorded as being within normal parameters. APTT and PT ratios did not predict thrombosis recurrence, while models using TEG (K, Angle, MA) and TGT (Peak, ETP) were able to predict it, highlighting a stronger clinical utility and non-interchangeability of the platforms between whole blood and plasma (viscoelastic test versus thrombin generation).

Inflammation/fibrin turnover predicted TEG positivity, but not TGT, suggesting a tendency for a bias towards positivity of viscoelastic results in an inflammatory context. Clinically, TEG/TGT should thus be targeted at the time of diagnosis and during treatment, taking into account the inflammation status. However, larger longitudinal studies are needed, with standardized sampling and broader screening of the cohort by exclusion criteria that include ancillary events that may induce thrombosis.

## Figures and Tables

**Table 1:** Patient characteristics included in the study (total participants *n* = 61).

Category	Number of Patients (*n*)	Percent of Total (%)
Thrombosis history	20	32.8
Cardiac involvement	37	60.7
Hepatic involvement	21	34.4
Renal involvement	34	55.7
Hypercoagulability by TEG	31	50.8
Hypercoagulability by TGT	25	41
Under therapy (daratumumab)	43	70.5
At initial diagnosis	18	29.5
DOACs	26	42.6
MAYO stage I	24	39.3
MAYO stage II	22	36.0
MAYO stage IIIA	9	14.7
MAYO stage IIIB	6	9.8
PALLADINI stage I	30	49.1
PALLADINI stage II	25	40.9
PALLADINI stage III	6	9.8

**Table 2:** Median, IQR, P95 and normal interval ranges of lab results.

	Test Type	Median	IQR (25–75%)	95% Percentile	Normal Interval Range
Biochemistry assays					
	ALBUMIN	3.6	2.8–9.8	4.9	3.5–5 g/dL
	κ	16	8.2–59	513	3.3–19.4 mg/L
	λ	23	12–48	3609	5.7–26.3 mg/L
	CRP	8	2.7–24	74	0.8–3.0 mg/L
Coagulation assays (no DOACs)					
	APTT-R	0.97	0.92–1.1	1.5	0.8–1.2
	PT	96	84–109	124	80–120%
	FX	98	78–156	144	60–140%
Coagulation assays					
	FG	490	396–570	713	200–400 mg/dL
	D DIMERS	224	132–457	1118	<500 ng/mL
TEG					
	R	5.3	4–6.7	7.8	2–8 min
	K	1.2	1–1.5	1.9	1–3 min
	ANGLE	73	68–75	80	55–78 degrees
	MA	68	64–75	80	51–69 mm
	G	10	9–15	20	4.6k–10.9k d/sec
TGT					
	LAG	3.7	2.8–5.1	11	min
	PEAK	175	110–281	470	nM
	TIME TO PEAK	6.7	5.5–7.7	12	min
	ETP	1127	793–1611	2169	nM/min

## Data Availability

All data can be made available by contacting our corresponding author.

## Abbreviations

The following abbreviations are used in this manuscript:

APTT-r	Activated partial thromboplastin time ratio
AUC	Area under the curve
CRP	C-reactive protein
DOACs	Direct anti coagulants
ETP	Endogenous thrombin potential
FG	Fibrinogen
FX	Coagulation factor X
IQR	Interquartile range
LCA	Light chain amyloidosis
MA	Maximum amplitude
NETs	Neutrophil extracellular traps
P95	95th Percentile
PPP	Platelet poor plasma
PT	Prothrombin Time
TEG	Thromboelastography
TGT	Thrombin Generation Time

## References

[1] Al Hamed R., Bazarbachi A.H., Bazarbachi A., Malard F., Harousseau J.-L., Mohty M. (2021). Comprehensive Review of AL amyloidosis: Some practical recommendations. Blood Cancer J..

[2] Feitosa V.A., Neves P.D.M.M., Jorge L.B., Noronha I.L., Onuchic L.F. (2022). Renal amyloidosis: A new time for a complete diagnosis. Braz. J. Med. Biol. Res..

[3] Merlini G., Seldin D.C., Gertz M.A. (2011). Amyloidosis: Pathogenesis and new therapeutic options. J. Clin. Oncol..

[4] Neculae G., Adam R., Jercan A., Bădeliță S., Tjahjadi C., Draghici M., Stan C., Bax J.J., Popescu B.A., Marsan N.A. (2024). Cardiac amyloidosis is not a single disease: A multiparametric comparison between the light chain and transthyretin forms. ESC Heart Fail..

[5] Palladini G., Milani P., Merlini G. (2020). Management of AL amyloidosis in 2020. Blood.

[6] Muchtar E., Grogan M., Aus dem Siepen F., Waddington-Cruz M., Misumi Y., Carroll A.S., Clarke J.O., Sanchorawala V., Milani P., Caccialanza R. (2025). Supportive care for systemic amyloidosis: International Society of Amyloidosis (ISA) expert panel guidelines. Amyloid.

[7] Nicol M., Siguret V., Vergaro G., Aimo A., Emdin M., Dillinger J.G., Baudet M., Cohen-Solal A., Villesuzanne C., Harel S. (2022). Thromboembolism and bleeding in systemic amyloidosis: A review. ESC Heart Fail..

[8] Müller C., Warth A. (2025). Light-Chain (AL) Amyloidosis as a Rare Cause of Upper Gastrointestinal Bleeding: A Case Report and Systematic Literature Review. Case Rep. Oncol..

[9] Axentiev A., Rozik M., Slama E., Setya V. (2023). Immunoglobulin Light Chain Amyloidosis Presenting as Inferior Vena Cava Thrombosis. Am. Surg..

[10] Himcinschi M.E., Uscatescu V., Gherghe G., Stoian I., Vlad A., Popa D.C., Coriu D., Anghel A. (2024). The Role of Neutrophil Extracellular Traps in the Outcome of Malignant Epitheliomas: Significance of CA215 Involvement. Diagnostics.

[11] Azevedo E.P.C., Guimarães-Costa A.B., Torezani G.S., Braga C.A., Palhano F.L., Kelly J.W., Saraiva E.M., Foguel D. (2012). Amyloid fibrils trigger the release of neutrophil extracellular traps (NETs), causing fibril fragmentation by NET-associated elastase. J. Biol. Chem..

[12] Paton E.L., Barisic S., Sabile J.M., Medvedova E. (2024). Acute deep vein thrombosis with concurrent new diagnosis of AL-amyloid-induced factor X deficiency. BMJ Case Rep..

[13] Rekhtina I.G., Khyshova V.A., Zozulya N.I., Dvirnyk V.N., Mendeleyeva L.P. (2023). Hemostasis disorders in patients with systemic AL-amyloidosis. Ter. Arkh..

[14] Rastoder E., Kamstrup P., Hedsund C., Jordan A., Sivapalan P., Rømer V., Falkvist F., Hamidi S., Bendstrup E., Sperling S. (2024). Thrombelastography and Conventional Coagulation Markers in Chronic Obstructive Pulmonary Disease: A Prospective Paired-Measurements Study Comparing Exacerbation and Stable Phases. Int. J. Mol. Sci..

[15] Kvisselgaard A.D., Wolthers S.A., Wikkelsø A., Holst L.B., Drivenes B., Afshari A. (2025). Thromboelastography or rotational thromboelastometry guided algorithms in bleeding patients: An updated systematic review with meta-analysis and trial sequential analysis. Acta Anaesthesiol. Scand..

[16] Gehlen R., Vandevelde A., de Laat B., Devreese K.M.J. (2023). Application of the thrombin generation assay in patients with antiphospholipid syndrome: A systematic review of the literature. Front. Cardiovasc. Med..

[17] Maxey-Jones C., Seelhammer T.G., Arabia F.A., Cho B., Cardonell B., Smith D., Leo V., Dias J., Shore-Lesserson L., Hartmann J. (2025). TEG^®^ 6s-Guided Algorithm for Optimizing Patient Blood Management in Cardiovascular Surgery: Systematic Literature Review and Expert Opinion. J. Cardiothorac. Vasc. Anesth..

[18] Hvas C.L., Larsen J.B., Adelborg K., Christensen S., Hvas A.-M. (2022). Dynamic Hemostasis and Fibrinolysis Assays in Intensive Care COVID-19 Patients and Association with Thrombosis and Bleeding-A Systematic Review and a Cohort Study. Semin. Thromb. Hemost..

[19] Lim H.Y., Donnan G., Nandurkar H., Ho P. (2022). Global coagulation assays in hypercoagulable states. J. Thromb. Thrombolysis.

[20] Favaloro E.J., Pasalic L. (2024). Routine Coagulation. Clin. Lab. Med..

[21] Asimakopoulos F., Hope C., Johnson M.G., Pagenkopf A., Gromek K., Nagel B. (2017). Extracellular matrix and the myeloid-in-myeloma compartment: Balancing tolerogenic and immunogenic inflammation in the myeloma niche. J. Leukoc. Biol..

[22] Kastritis E., Palladini G., Minnema M.C., Wechalekar A.D., Jaccard A., Lee H.C., Sanchorawala V., Gibbs S., Mollee P., Venner C.P. (2021). Daratumumab-Based Treatment for Immunoglobulin Light-Chain Amyloidosis. N. Engl. J. Med..

[23] Hughes M.S., Lentzsch S. (2023). Safety and Efficacy of Subcutaneous Daratumumab in Systemic AL Amyloidosis. Ther. Clin. Risk Manag..

[24] Winter W.E., Flax S.D., Harris N.S. (2017). Coagulation Testing in the Core Laboratory. Lab. Med..

[25] Johnson E.D., Schell J.C., Rodgers G.M. (2019). The D-dimer assay. Am. J. Hematol..

[26] Burton A.G., Jandrey K.E. (2020). Use of Thromboelastography in Clinical Practice. Vet. Clin. N. Am. Small Anim. Pract..

[27] Josset L., Rezigue H., Dargaud Y. (2025). Thrombin Generation Assay to Support Hematologists in the Era of New Hemophilia Therapies. Int. J. Lab. Hematol..

[28] O’Leary J.G., Greenberg C.S., Patton H.M., Caldwell S.H. (2019). AGA Clinical Practice Update: Coagulation in Cirrhosis. Gastroenterology.

[29] Liang F., Wang Y. (2021). Coronary heart disease and atrial fibrillation: A vicious cycle. Am. J. Physiol. Heart Circ. Physiol..

[30] Kearney D., Leonberg-Yoo A., Cohen R. (2023). Frequent vascular access thrombosis in a patient with end stage kidney disease on hemodialysis. Semin. Dial..

[31] Ferdinande K., Raevens S., Decaestecker J., De Vloo C., Seynhaeve L., Hoof L., Verhelst X., Geerts A., Devreese K.M.J., Degroote H. (2024). Unravelling the coagulation paradox in liver cirrhosis: Challenges and insights. Acta Clin. Belg..

[32] Sabatino A., Regolisti G., Karupaiah T., Sahathevan S., Sadu Singh B.K., Khor B.H., Salhab N., Karavetian M., Cupisti A., Fiaccadori E. (2017). Protein-energy wasting and nutritional supplementation in patients with end-stage renal disease on hemodialysis. Clin. Nutr..

[33] Fontana M., Ioannou A., Cuddy S., Dorbala S., Masri A., Moon J.C., Singh V., Clerc O., Hanna M., Ruberg F. (2025). The Last Decade in Cardiac Amyloidosis: Advances in Understanding Pathophysiology, Diagnosis and Quantification, Prognosis, Treatment Strategies, and Monitoring Response. Cardiovasc. Imaging.

[34] Fotiou D., Theodorakakou F., Spiliopoulou S., Gavriatopoulou M., Migkou M., Kanellias N., Eleutherakis-Papaiakovou E., Malandrakis P., Dialoupi I., Roussou M. (2024). Thrombotic and bleeding complications in patients with AL amyloidosis. Br. J. Haematol..

[35] Wechalekar A.D., Sanchorawala V. (2022). Daratumumab in AL amyloidosis. Blood.

[36] Douxfils J., Ageno W., Samama C.-M., Lessire S., Ten Cate H., Verhamme P., Dogné J.-M., Mullier F. (2018). Laboratory testing in patients treated with direct oral anticoagulants: A practical guide for clinicians. J. Thromb. Haemost..

[37] Singh A.D., Mucha S.R., Lindenmeyer C.C. (2022). Cirrhotic coagulopathy: A rebalanced hemostasis. Cleve. Clin. J. Med..

[38] Lancé M.D. (2015). A general review of major global coagulation assays: Thrombelastography, thrombin generation test and clot waveform analysis. Thromb. J..

[39] Carll T. (2023). Viscoelastic Testing Methods. Adv. Clin. Chem..

[40] Valke L.L.F.G., Rijpma S., Meijer D., Schols S.E.M., van Heerde W.L. (2022). Thrombin generation assays to personalize treatment in bleeding and thrombotic diseases. Front. Cardiovasc. Med..

